# A composite lymphoma combining a Hodgkin lymphoma and a marginal zone lymphoma transformed into a diffuse large B‐cell lymphoma

**DOI:** 10.1002/ccr3.1841

**Published:** 2018-10-22

**Authors:** Claire Auditeau, Olivier Lambotte, Joffrey Feriel, Thierry Lazure, Ali Turhan, Cédric Aumont

**Affiliations:** ^1^ Division of Hematology, Centre Hospitalier Universitaire Université Paris Sud 11 Le Kremlin Bicêtre France; ^2^ Department of Internal Medecine, Centre Hospitalier Universitaire Université Paris Sud 11 Le Kremlin Bicêtre France; ^3^ INSERM U935 Villejuif France; ^4^ Department of Anatomopathology, Centre Hospitalier Universitaire Université Paris Sud 11 Le Kremlin Bicêtre France

**Keywords:** composite lymphoma, Hodgkin lymphoma, non‐Hodgkin lymphoma, Richter syndrome

## Abstract

Composite lymphoma is defined as the occurrence of two or more distinct lymphoma types in a single anatomic site. We report a case of Richter syndrome with both Hodgkin lymphoma and non‐Hodgkin lymphoma in the bone marrow. This diagnostic was suspected because of discrepancies between histological and cytological results.

## 1 INTRODUCTION

We present the case of a patient suffering from a composite lymphoma combining a Hodgkin lymphoma and a diffuse large B‐cell lymphoma (DLBCL) revealed by a hemophagocytic syndrome. Bone marrow analysis revealed a surprising discrepancy between the results of the bone marrow aspirate which suggested the diagnosis of DLBCL and that of the bone marrow biopsy, in favour of a marginal zone lymphoma associated with a classic Hodgkin lymphoma (HL). The patient was treated for the Hodgkin lymphoma component of his malignancy. After two cycles of ABVD, a CD10+ lymphocyte population remained, compatible with a DLBCL. The final diagnosis was a Richter syndrome with transformation of a marginal zone lymphoma into a DLBCL, associated with a Hodgkin lymphoma.

## 2 CASE REPORT

**Figure 1:**
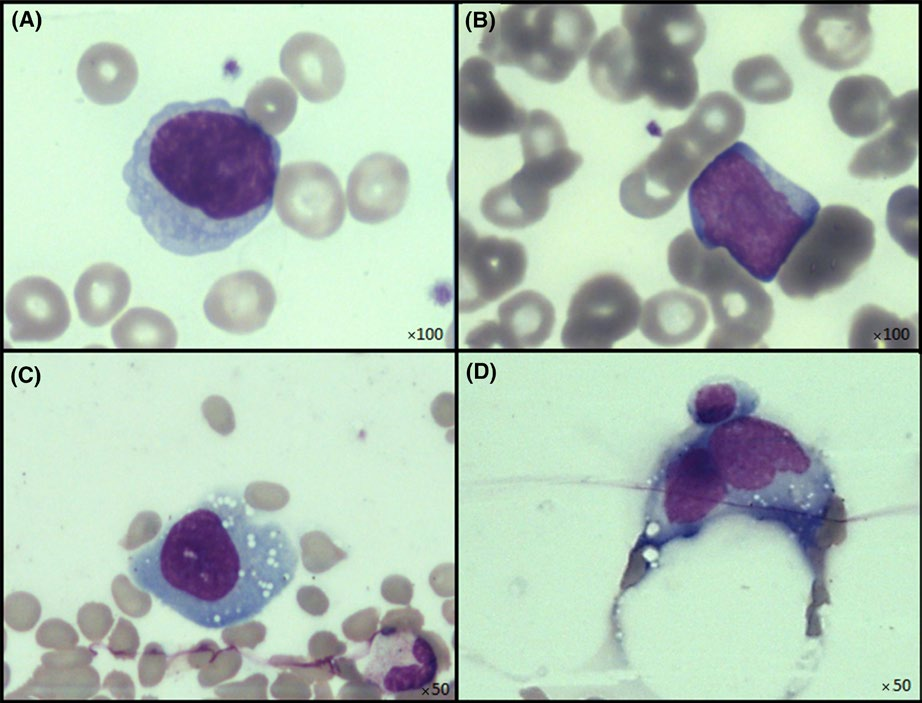
Cytological analysis of the blood smear. A and B, Medium large lymphocytes, with a slightly basophilic cytoplasm, a regular nucleus and mature chromatin with nucleoli. C and D, Rare atypical cells with a basophilic cytoplasm, irregular nucleus and vacuoli

A 70‐year‐old man was addressed to the emergency department with a suspicion of prostatitis. His medical history included urethral stricture requiring self‐urinary catheterization, and kidney failure. Anamnesis and clinical examination revealed an alteration of general state (asthenia, anorexia and status 2 of WHO performance) associated with fever at 39.6°C and sweats that had lasted for 2 weeks. Two different antibiotic treatments (Sulfamethoxazol‐Trimethoprim first, then Amoxicillin) were given, without any improvement. The initial blood count showed a anaemia (haemoglobin at 12.1 g/dL), neutrophilia (neutrophils at 16.8 × 10^9^/L), and lymphocytosis (lymphocytes at 10.9 × 10^9^/L). Blood smear showed medium large lymphocytes with regular nucleus and nucleoli (Figure 1A,B), and rare atypical large lymphocytes (Figure 1C,D). Cytobacteriological examination of urine was negative. An abdominal computerized tomography (CT) scan revealed a hepato‐splenomegaly associated with coeliac and mesenteric adenopathy. The patient was admitted to the hospital in order to explore a probable hematologic malignancy.

**Figure 2:**
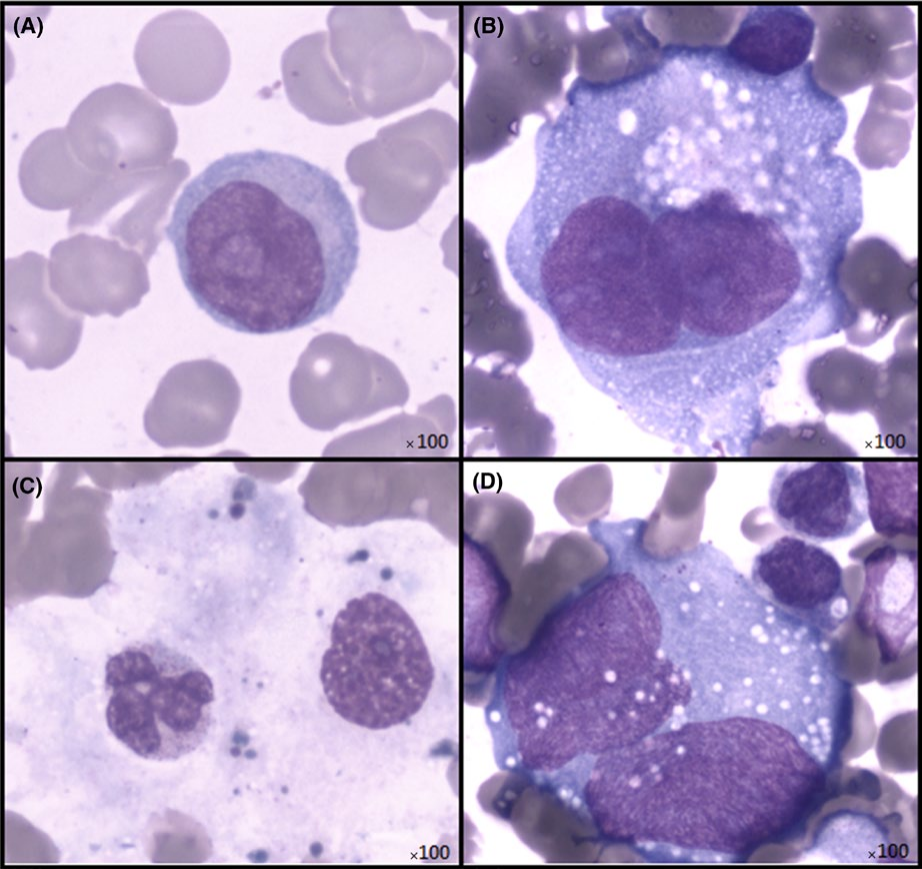
Cytological analysis of bone marrow. A, Bone marrow aspirate performed during the initial workup showing a large lymphocyte with basophilic cytoplasm, regular nucleus and nucleoli. B, The same aspirate revealing atypical large cells with intense basophilic cytoplasm, vacuoles, irregular nucleus, decondensed chromatin, and multiple nucleoli, thought to be a Sternberg‐like cell. C, Second bone marrow aspirate: evidence of hemophagocytosis confirming the persisting hemophagocytic syndrome. D, Second bone marrow aspirate: Reed‐Sternberg cell

**Figure 3:**
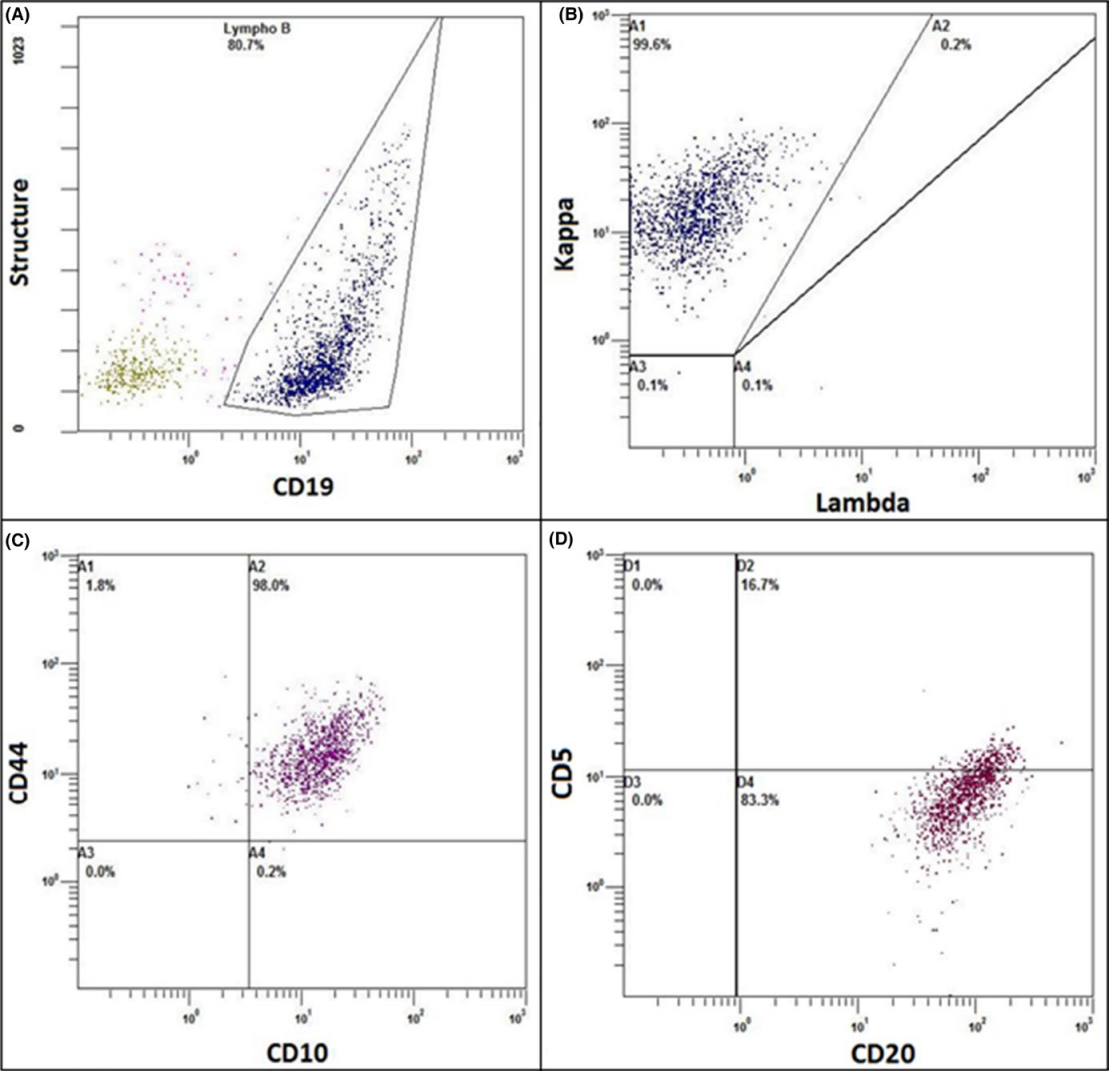
Flow cytometry plots. A, Heterogeneous structure of the B‐cell population. B, Monotypic Kappa +cell population. C and D, CD 10+/CD20+/CD5− B‐cell population

A biochemistry panel showed highly increased levels of blood ferritin levels at 51 681 pmol/L, associated with hypertriglyceridemia at 3 mmol/L and discrete cytolysis, leading to a strong suspicion of hemophagocytic syndrome, with a Fardet^1^ probability score of 88.2%. A marrow aspiration was carried out. There was a major infiltration of bone marrow with macrophages but no clear diagnosis of hemophagocytosis could be performed. Nearly, half of the lymphocytes observed had morphology compatible with a DLBCL including large lymphocytes, with a basophilic cytoplasm; a regular nucleus and a compacted chromatin including a nucleolus (Figure 2A). Cytological analysis revealed also the presence of large atypical cells with intense basophilic cytoplasm containing vacuoles, with irregular nuclei, and some binucleated cells with decondensed chromatin and multiple nucleoli (Figure 2B). These cells were thought to be Reed‐Sternberg‐like cells which may be observed in patients with DLBCL.^2^, ^3^, ^4^ Immunophenotypic analysis performed on the bone marrow aspirate demonstrated the presence of a heterogeneous B population monotypic kappa (Figure 3A,B), with a strong expression of CD20 and expression CD10, and negative for CD5 and CD38 expression (Figure 3C,D). Cytology and immunophenotyping were in favor of a diffuse large B‐cell lymphoma (DLBCL), but were insufficient to make a precise diagnostic. Moreover, virological tests were performed on a whole blood sample and revealed the presence of Epstein‐Barr virus (EBV) DNA with a viral load of 4.24 log_10_ copies/mL.

**Figure 4:**
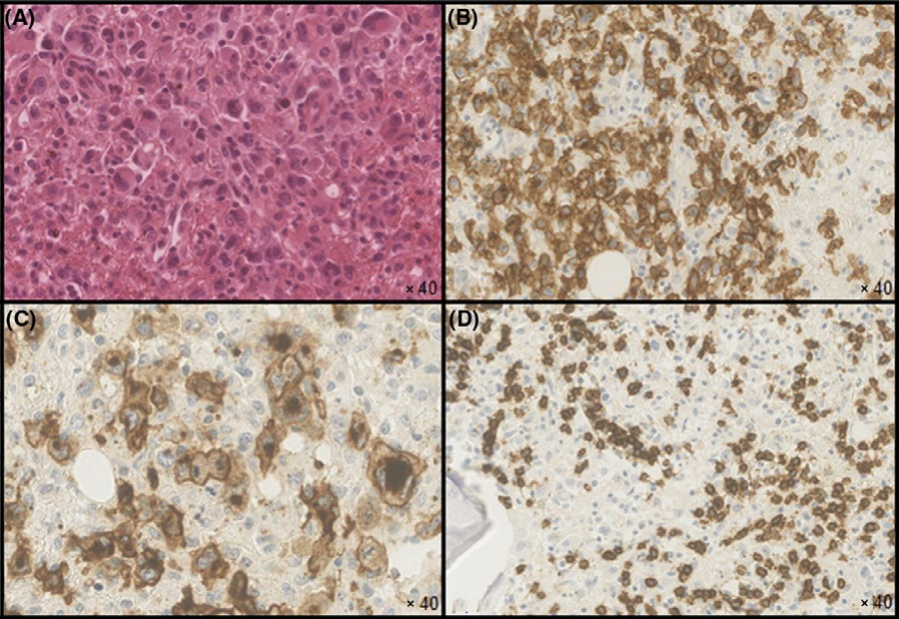
Analysis of bone marrow biopsy. A, Reed‐Sternberg cells with hematoxylin‐eosin saffron coloration. B, CD30 tumour cells composing the Hodgkin lymphoma. C, CD15‐positive tumour cells composing the Hodgkin lymphoma. D, Interstitial and intrasinusoidal infiltrate of small lymphocytes CD20+ CD10− CD5− in favour of a marginal zone lymphoma

A bone marrow biopsy was performed. It revealed typical Reed‐Sternberg cells (Figure 4A) suggesting the diagnosis of Hodgkin lymphoma, associated with large numbers of macrophages typical of a hemophagocytic syndrome. An interstitial and intrasinusoidal infiltrate of small lymphocytes was also observed. Immunohistochemistry tests highlighted a population of CD30+ (Figure 4B), CD15+ (Figure 4C), LMP1−, CD20−, CD79a−, CD3−, CD5−, and ALK− cells. The use of anti‐CD20 antibodies made possible to identify a contingent of CD5− and CD10− cells, compatible with a marginal zone lymphoma (Figure 4D). Surprisingly, the population of CD10+ lymphocytes detected with the bone marrow immunophenotyping could not be detected in the bone marrow biopsy.^5^

The final diagnosis was a Richter syndrome transforming a marginal zone lymphoma into a DLBCL associated with a stage IV lymphocyte‐depleted classic Hodgkin lymphoma revealed by a hemophagocytic syndrome.

Before the results of the biopsy, the patient received one cycle of COP chemotherapy (Cyclophosphamide, Vincristine and Prednisone) aiming to treat his DLBCL. When the diagnosis of HL was firmly established, he was treated by ABVD chemotherapy (Doxorubicin, Bleomycin, Vinblastine, and Dacarbazine) associated with VP16 (Etoposide) to treat the hemophagocytic syndrome. After two cycles of ABVD chemotherapy, a slight lymphocytosis remained. A second immunophenotyping revealed a persistent CD20+ CD10+ lymphocyte population. The patient was then treated by three cycles of R‐CHOEP chemotherapy (Rituximab, Cyclophosphamide, Doxorubicin, Vincristine, Etoposide, and Prednisone). The initial response to this therapy was satisfactory, with improvement of the hemophagocytic syndrome and decrease in the ferritin levels from 29 211 to 9437 pmol/L. After the third R‐CHOEP cycle, the patient was admitted to the hospital because of a sepsis triggered by a urinary infection. His general state quickly worsened, and a second bone marrow aspirate revealed a relapse of the hemophagocytic syndrome (Figure 2C) and a persistence of the Hodgkin lymphoma (Figure 2D). Despite antibiotic treatment and chemotherapy with high doses of Etoposide, Rituximab, and Cisplatin, there was no amelioration and a multiple organ failure led to the patient’s decease.

## 3 DISCUSSION

The occurrence of concomitant classic Hodgkin lymphoma and a variety of B‐cell non‐Hodgkin lymphoma (NHL) in the same tissue defines the diagnostic criteria for composite lymphomas. Composite lymphomas have been described in the literature^6^, ^7^, ^8^, ^9^, ^10^, ^11^ with association of: Hodgkin lymphoma with gastric mucosa‐associated lymphoid tissue (MALT) lymphoma,^6^ splenic marginal zone lymphoma,^7^, ^8^ follicular lymphoma,^9^ chronic lymphocytic leukaemia,^10^ or large B‐cell lymphoma.^11^

To the best of our knowledge, there is no case report of a Hodgkin lymphoma with medullary involvement and a DLBCL.

Numerous studies aimed to identify a common B‐cell precursor in patients with both Hodgkin lymphoma and NHL. Braüninger et  al^12^ discovered somatic mutations carried by V genes (rearranged immunoglobulin variable‐region genes) shared by Reed‐Sternberg and NLH cells. These results gave rise to the hypothesis of a common germinal‐centre B‐cell precursor. More recently, the case of a 66‐year‐old woman presenting sequentially a splenic marginal zone lymphoma, a Hodgkin lymphoma, and a DLBCL (thought to be the evolution of the MZL) was reported (Sara Alvaro et  al case report 2016^13^). The analysis of clonally rearranged IGH genes revealed the three histological types of lymphoma had the exact same clonal IGH rearrangement.

Composite lymphoma diagnosis represents a challenge due to the heterogeneity of clinical, biological, and histological aspects of the disease. It has to be suspected when a discrepancy occurs between the different histological tests carried out. In the case reported here, the results of the bone marrow aspirate and the bone marrow biopsy were discordant. Firstly, each exploration test limits have to be considered. For instance, CD10+ lymphocytes were not detected by immunohistochemistry tests on the biopsy, whereas this lymphocyte population was found on the bone marrow immunophenotyping. It has already been described that the antibodies used for these two tests target different epitopes,^14^ and that the immunohistochemistry is less sensible than the immunophenotyping because the epitopes targeted in this technique are more likely to disappear when the patient develops a DLBCL. As a second example, it is exceptional to see a Hodgkin cell or a Reed‐Sternberg cell on a bone marrow smear. This technique is known to be less sensible than the bone marrow biopsy to detect these types of cells.^5^ Moreover, when a DLBCL is suspected, it has already been described that Sternberg‐like cells may be observed on the bone marrow smear.^2^, ^3^, ^4^ Secondly, it is important to do repeated tests in various sites in order to increase the sensitivity. A lymph node aspirate or biopsy could have given further information, but was impossible due to the location of the patient’s lymphadenopathy and his frail condition.

In summary, in the case reported here, the correct diagnosis was possible by correlating clinical, cytological, and pathological features of the patient’s history allowing to identify this case as a Richter syndrome resulting from the transformation of a marginal zone lymphoma into a DLBCL associated with a Hodgkin lymphoma. As a result, the treatment was adapted to target both components of this composite lymphoma.

## CONFLICT OF INTEREST

None declared.

## AUTHOR CONTRIBUTION

AC: performed the cytologic and phenotypic analysis for diagnosis, synthesized the medical record, and wrote the paper. LO: performed the clinical care of the patient in the in‐patient setting and wrote the paper. FJ: supervised the laboratory steps for cytological diagnosis. LT: performed anatomopathologic analyses. TA: provided the scientific and bibliographic features and wrote the paper. AC: supervised the cytological analyses and phenotypic analysis, synthesized the entire clinical history, and wrote the paper.

## References

[1] Fardet L , Galicier L , Lambotte O , et al. Development and validation of the HScore, a score for the diagnosis of reactive hemophagocytic syndrome. Arthritis Rheumatol. 2014;66(9):2613‐2620. 24782338 10.1002/art.38690

[2] Sancheti S , Arora N , Sawaimoon S , Chakrapani A . Follicular lymphoma evolving into diffuse large B‐cell lymphoma with Reed‐Sternberg like cells. Indian J Pathol Microbiol. 2015;58(2):261‐262. 25885156 10.4103/0377-4929.155354

[3] Gomez‐Gelvez JC , Smith LB . Reed‐Sternberg–like cells in non‐Hodgkin lymphomas. Arch Pathol Lab Med. 2015;139(10):1205‐1210. 26414463 10.5858/arpa.2015-0197-RAI

[4] Wang E , Papavassiliou P , Sebastian S . A malignant lymphoma with histological features and immunophenotypic profile intermediate between EBV‐positive diffuse large B‐cell lymphoma and EBV‐positive classical Hodgkin lymphoma in a 67‐year‐old female: a "gray zone" lymphoma associated with Epstein‐Barr virus in the elderly. Pathol Res Pract. 2012;208(6):363‐367. 22572037 10.1016/j.prp.2012.04.003

[5] Toi PC , Varghese RG , Rai R . Comparative evaluation of simultaneous bone marrow aspiration and bone marrow biopsy: an institutional experience. Indian J Hematol Blood Transfus. 2010;26(2):41‐44. 21629634 10.1007/s12288-010-0010-x PMC3002064

[6] Zeidan A , Faltas B , Forde P , Subhawong A , Bello C , Bolaños‐Meade J . Sequential occurrence of a splenic marginal zone lymphoma, extranodal MALT lymphoma, and Hodgkin lymphoma. Clin Lymphoma Myeloma Leuk. 2013;13(4):496‐498. 23747079 10.1016/j.clml.2013.04.004 PMC4659405

[7] Elmahy H , Hawley I , Beard J . Composite splenic marginal zone lymphoma and classic Hodgkin lymphoma—an unusual combination. Int J Lab Hematol. 2007;29(6):461‐463. 17988302 10.1111/j.1365-2257.2006.00859.x

[8] Harada S , Kalla H , Balasubramanian M , Brodsky I , Gladstone D , Hou JS . Classical Hodgkin lymphoma concurrently evolving in a patient with marginal zone B‐cell lymphoma of the spleen. Ann Diagn Pathol. 2008;12(3):212‐216. 18486899 10.1016/j.anndiagpath.2006.12.005

[9] Gonzalez CL , Medeiros LJ , Jaffe ES . Composite lymphoma – a clinicopathologic analysis of nine patients with Hodgkin's lymphoma and B‐cell NHL. Am J Clin Pathol. 1991;96:81‐89. 2069139 10.1093/ajcp/96.1.81

[10] Hansmann ML , Fellbaum C , Hui PK , Lennert K . Morphological and immunohistochemical investigation of non‐Hodgkin's lymphoma combined with Hodgkin's disease. Histopathology. 1989;15:35‐48. 2548946 10.1111/j.1365-2559.1989.tb03039.x

[11] Bellan C , Lazzi S , Zazzi M , et al. Immunoglobulin gene rearrangement analysis in composite hodgkin disease and large B‐cell lymphoma: evidence for receptor revision of immunoglobulin heavy chain variable region genes in Hodgkin‐Reed‐Sternberg cells? Diagn Mol Pathol. 2002;11(1):2‐8. 11854595 10.1097/00019606-200203000-00002

[12] Brauninger A , Hansmann ML , Strickler JG , et al. Identification of common germinal‐center B‐cell precursors in two patients with both Hodgkin's disease and non‐Hodgkin's lymphoma. N Engl J Med. 1999;340:1239‐1247. 10210707 10.1056/NEJM199904223401604

[13] Xu Y , McKenna RW , Kroft SH . Comparison of multiparameter flow cytometry with cluster analysis and immunohistochemistry for the detection of CD10 in diffuse large B‐Cell lymphomas. Mod Pathol. 2002;15(4):413‐419. 11950915 10.1038/modpathol.3880539

[14] Alonso‐Alvarez S , Redondo‐Guijo A , Blanco Ó , et al. Lymphoma heterogeneity: three different histological pictures and one unique clone. Case Rep Hematol. 2016;2016:3947510. 27867670 10.1155/2016/3947510 PMC5102716

